# Effects of High-Intensity Resistance Training on Fitness and Fatness in Older Men With Osteosarcopenia

**DOI:** 10.3389/fphys.2020.01014

**Published:** 2020-08-27

**Authors:** Wolfgang Kemmler, Matthias Kohl, Michael Fröhlich, Klaus Engelke, Simon von Stengel, Daniel Schoene

**Affiliations:** ^1^Institute of Medical Physics, Friedrich-Alexander University of Erlangen-Nürnberg, Erlangen, Germany; ^2^Faculty Medical and Life Sciences, University of Furtwangen, Villingen-Schwenningen, Germany; ^3^Department of Sports Science, University of Kaiserslautern, Kaiserslautern, Germany; ^4^Department of Medicine III, Friedrich-Alexander University of Erlangen-Nürnberg, University Hospital Erlangen, Erlangen, Germany

**Keywords:** exercise, sarcopenia, body composition, strength, older men

## Abstract

To date, there has been no study on the long-term effects of resistance exercise on sarcopenia and obesity indices for people with sarcopenia. The present study thus aimed to determine the effect of 18 months of periodized, high-velocity/intensity/effort progressive resistance training (HIT-RT) on body composition and strength in older men with osteosarcopenia. Using a single-blind, two-group parallel design, 43 community-dwelling men, 72 years and older, with osteopenia and sarcopenia in Erlangen-Nürnberg, Germany, were randomly assigned to two study arms by drawing lots: (1) an exercise group that conducted a consistently supervised periodized high-velocity/intensity/effort protocol (HIT-RT; *n* = 21) on machines twice a week for 18 months or (2) a control group (CG; *n* = 22) that maintained their physical activity/exercise habits. Both groups were supplied with protein, cholecalciferol, and calcium according to current recommendations. The study outcomes were lean body mass (LBM), total and abdominal body fat as determined by dual-energy X-ray absorptiometry and maximum hip/leg extensor strength as assessed on an isokinetic leg press at baseline and after 8, 12, and 18 months of follow-up. The intention-to-treat principle and multiple imputation were applied to calculated study outcomes. After 18 months of HIT-RT, altogether five participants were lost to follow up (HIT-RT: *n* = 2, CG: *n* = 3). The attendance rates (95%) for HIT-RT were high; relevant adverse effects were not observed. Significant effects (i.e., differences between HIT-RT vs. CG) in favor of HIT-RT were determined for LBM (+1.73 kg, 95% CI: +1.13 to +2.32 kg), total body fat mass (−2.44 kg, 95% CI: −1.28 to 3.60 kg), abdominal body fat percentage (−2.68, 95% CI: −1.70 to −3.66), and maximum hip/leg extensor strength (+533 N, 95% CI: +397 to +670 N; all *p* < 0.001). Even after adjusting for multiple testing, all effects remained significant. Of note, after 8 months of HIT-RT, only slight (LBM and fat indices) to moderate (maximum strength) ongoing effects were observed. Carefully introduced, continuously supervised HIT-RT is an effective, attractive, feasible, and safe method to improve body composition and muscle strength in older community-dwelling men with sarcopenia. However, even when consequently applying principles of exercise intensity progression within the RT protocol, only slight further positive changes were observed after 8 months of exercise.

## Introduction

A large number of studies have found positive effects of dynamic resistance exercise training (DRT), with or without nutritional intervention, on body composition and physical fitness in older adults with sarcopenia or sarcopenic obesity (SO; review in Csapo and Alegre, 2016; Liao et al., 2017b; Martinez-Amat et al., 2018; Vlietstra et al., 2018; Beckwee et al., 2019; Hsu et al., 2019). Hereby the majority of exercise trials address fat-free mass, lean body mass (LBM), and muscle strength, while the effects of isolated DRT protocols on total or abdominal body fat are rarely evaluated. However, considering the relevance of total and abdominal body fat for cardiometabolic diseases (Amato et al., 2013), particularly in people with low muscle mass (Terada et al., 2017), this topic should be addressed with more emphasis (Lee et al., 2019). Furthermore, the intervention period of studies focusing on people with sarcopenia or SO averages between 10 and 26 weeks (Csapo and Alegre, 2016; Liao et al., 2017b; Martinez-Amat et al., 2018; Vlietstra et al., 2018; Beckwee et al., 2019; Hsu et al., 2019), i.e., periods that do not allow the full determination of DRT effects, particularly on body fat indices. Another problem that hinders the broad implementation of DRT protocols in the community might be the fact that most current DRT protocols focus on time-consuming multiple-set DRT applied three times per week, an approach that collides with the limited enthusiasm of most (older) people to frequently attend exercise classes (Carlson et al., 2010; DESTATIS, 2016).

The present study focuses on the effect of time-effective, high-intensity DRT (HIT-RT) in community-dwelling men, 72 years and older, with osteosarcopenia. In the present contribution, we focus on changes of body composition parameters and strength indices. In summary, we aimed to verify four primary hypotheses *en bloc*. We hypothesized that HIT-RT significantly improves (1) lean body mass, (2) total abdominal fat, (3) abdominal body fat, and (4) increases maximum isokinetic hip/leg-extensor strength (MILES) compared to a non-training control group (CG). In parallel, we focus on the ongoing effects of periodized HIT-RT on the parameters listed above. Our experimental hypothesis was thus that our progressive exercise protocol generates ongoing effects on LBM, total fat mass, abdominal fat percentage, and MILES during the intervention period.

## Materials and Methods

The Franconian Osteopenia and Sarcopenia Trial (FrOST) was an 18-month exercise trial with a balanced parallel-group design with community-dwelling men, 72 years and older, with low muscle and bone masses (i.e., osteosarcopenia). The project was initiated and conducted by the Institute of Medical Physics, University of Erlangen-Nürnberg (FAU), Germany, and was approved by the FAU Ethics Committee (number 67_15b and 4464b) and the Federal Bureau of Radiation Protection (BfS, number Z 5 – 2246212-2017-002). The FrOST study fully complies with the Helsinki Declaration (World_Medical_Association, 2013). After receiving detailed information about all study aspects, the study participants gave their written informed consent. The present publication focuses on body composition and strength effects after 18 months of continuous exercise.

### Participants

**Figure 1:**
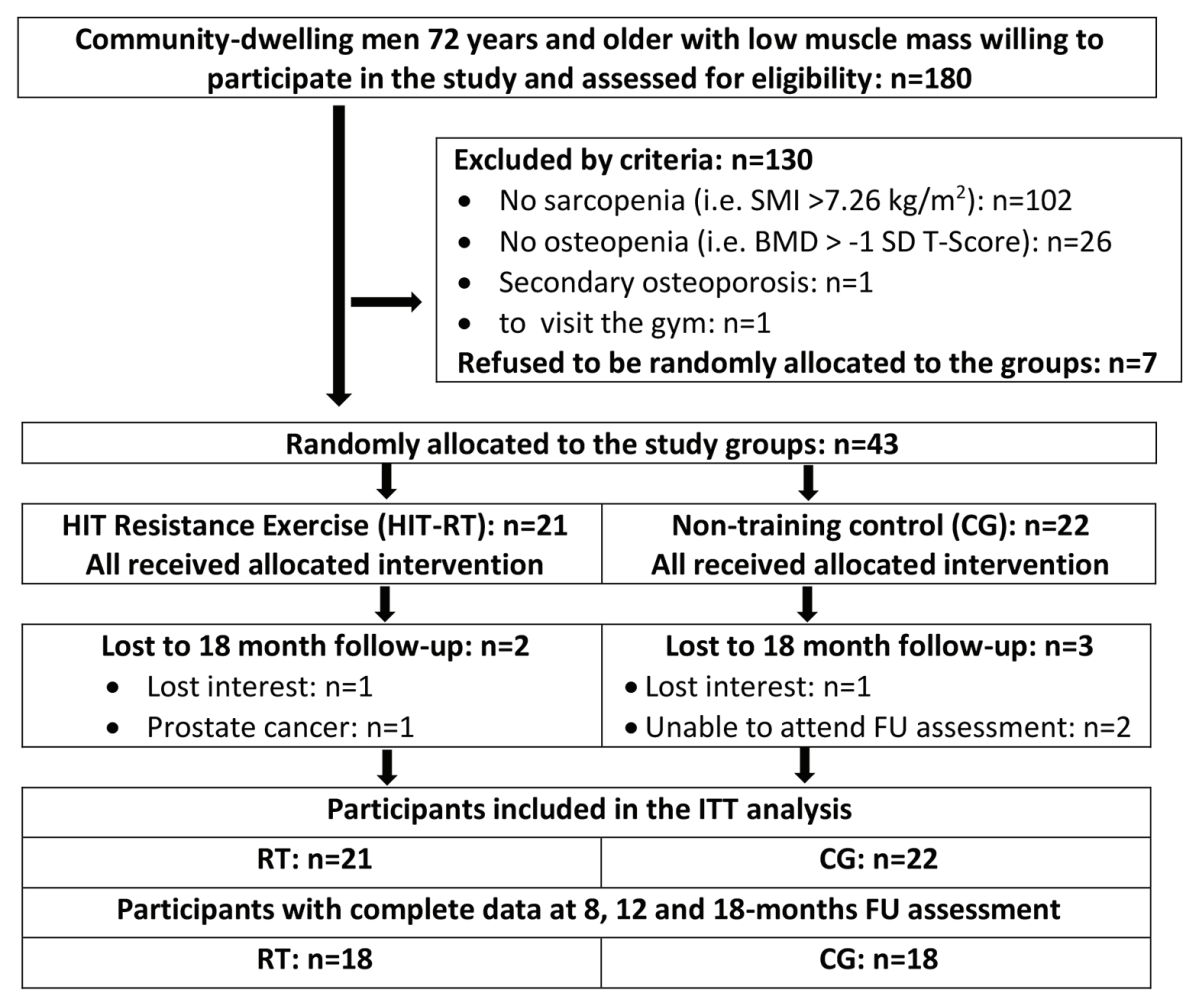
Participant flow through the study.

The FrOST recruitment procedure took place between March and May 2018 and has been described in detail in previous contributions (Lichtenberg et al., 2019; Kemmler et al., 2020b). Thus, only a brief summary will be provided here. One hundred eighty men, 72 years and older, of lowest skeletal muscle mass (SMI) index (see “Methods”) quartile (*n* = 242) of the epidemiologic Franconian Sarcopenic Obesity (FranSO) study (*n* = 965; Kemmler et al., 2017a, 2019) were contacted and participated in the 24-month follow-up (FU). Only community-dwelling men with morphologic sarcopenia [i.e., (low) skeletal muscle mass index ≤7.26 kg/m^2^; Baumgartner et al., 1998; Cruz-Jentoft et al., 2010] and osteopenia/osteoporosis at the lumbar spine or the proximal femur [i.e., bone mineral density (BMD) <−1 SD T-Score; WHO, 1994] were included (Figure 1). Men (a) with secondary osteoporosis, (b) with a history of hip fracture, (c) who had (osteo)anabolic and anti-resorptive pharmaceutic therapy, (d) who had glucocorticoid therapy >7.5 mg/day, (e) with diseases and health problems that prevent HIT-RT on machines, (f) who had resistance exercise (>60 min/week) during the last 5 years, (g) who had alcohol abuse >60 g/day ethanol, and (h) who had absence >2 weeks during the intervention period were excluded. A further seven participants refused to be randomly allocated to the groups. Finally, 43 eligible men willing to accept the randomization procedure were randomly assigned to HIT-RT (*n* = 21) and non-exercising control (*n* = 22) groups. Figure 1 shows the summarized participant flow through the study.

### Randomization Procedures

Stratified for SMI, the men allocated themselves to the HIT-RT or CG by drawing lots placed in small opaque capsules (“kinder egg,” Ferrero, Italy). A person not involved in the present project supervised the procedure. Neither researchers nor participants knew the allocation beforehand (allocation concealment). Next, the participants were enrolled and instructed by the primary investigator about the further procedure and expected compliance related to their study status.

### Blinding

The outcome assessors and test assistants were not aware of the participants’ exercise status (HIT-RT or CG) and were not allowed to ask either.

### Study Procedure

FrOST aimed to determine the effect of isolated HIT-RT on body composition and strength. Nevertheless, all the participants were supplied with whey protein, cholecalciferol, and calcium according to recent recommendations (Bauer et al., 2013; DVO, 2017), and they were all asked to maintain their dietary and physical activity habits.

### Interventions

#### Resistance Exercise

The HIT-RT protocol of FrOST has been reported in detail in previous articles (Kemmler et al., 2020a,b); thus, only a brief description will be given here. The exercise intervention focused exclusively on resistance exercise on machines (MedX, Ocala, FL, USA) without any other type of exercise, be it in parallel to the intervention or within warm-up or cool-down. The participants consistently exercised two times/week in a well-equipped gym (Kieser Training, Erlangen, Germany); in cases of temporary inability (holidays and illness), they were allowed to exercise three times in the week before and/or after. The consistently supervised HIT-RT was defined as single-set exercise training with high intensity and effort (Gießing, 2008). Exercise intensity was prescribed by a given range of repetitions (reps)/set (i.e., 5–7) and the corresponding set endpoint (Steele et al., 2017; “effort”) specified in “non-repetition maximum” (nRM), “self-determined repetition maximum (RM), and work to momentary failure (MF). Twelve to 14 exercises/session, taken from a pool of 18 exercises (calf raises, leg press, extension, leg curls, adduction, abduction, hip extension, latissimus front pulleys, pull-overs, seated rowing, back extension, inverse fly, bench press, military press, lateral raises, butterfly with extended arms, crunches, and lateral crunches), were applied. All exercises were consistently conducted using (nearly) the full range of motion and at varying movement velocity. All the participants of the HIT-RT group were provided with detailed training logs that prescribed exercises, number of repetitions (reps), movement velocity, and absolute exercise intensity (“effort”).

The 18-month HIT-RT was structured into eight periods of 8–12 weeks, with progressively increasing intensity/effort using intensifying strategies (Gießing, 2008). Apart from the initial familiarization and conditioning phase 1, each period was organized in linearly periodized mesocycles of 4 weeks, with each 4th week as a low-intensity/regeneration week. Before each phase, the participants were informed in detail about the aims and the procedure of the new training period in joint meetings.

During phase 1, we applied sets of eight to 15 reps without specific intensity prescription. Time under tension (TUT)/rep was specified as 2 s concentric, 1 s isometric, and 2 s eccentric (2s–1s–2s), with 90–120 s of rest pauses between the sets. In phase 2, we introduced the repetition in reserve approach (Zourdos et al., 2016). The participants were requested to choose a load that ensured a repetition maximum −1 rep (five to 10 reps) or −2 reps (10–18 reps). Movement velocity varied between the sessions (TUT: 4s–1s–4s to 1s–1s–2s/rep), with rest pauses of 90–120 s. In phase 3, we introduced (1) explosive movement during the concentric phase and (2) the RM approach (Steele et al., 2017) for sets ≤10 reps. From phase 3 on, up to one-third of the sets/sessions were conducted with an explosive movement, however never to RM. Rest pauses were 90–120 s. In phase 4, we introduced the superset approach. Four blocks of two or three exercises performed in a row addressed either related muscle groups or agonist/antagonist. Rest pauses were specified as ≈60 s within a superset block and 2 min after the superset. In phase 5, three superset blocks were intensified by drop sets. After work to RM (≤10 reps) or RM-1-2 rep (>10 reps), the participants decreased the load by 10–20% and immediately worked again to RM or RM-1 reps. Rest pauses averaged ≈60 s within and 2 min after the supersets. In phase 6, we (participants and investigators) decided to test the momentary failure approach (inability to realize the concentric phase of the current rep). However, after a negative feedback (“too hard”), we reverted to the RM approach. During the last 16 weeks of the intervention (phases 7 and 8), we did not introduce further significant changes. The only novelty was that the load was deceased twice within the drop set (particularly in the 3rd week of the 4-week mesocycle).

In summary, we applied a periodized HIT-RT with intensifying strategies (however without MF), varying relative (60–85% 1RM) and absolute (nRM to RM) exercise intensities, and alternating movement velocity (explosive-slow)/time under load/rep (3–9 s).

### Protein Supplementation

The participants were provided with whey protein powder (Active PRO80, inkospor, Roth, Germany) based on 4-day dietary protocols to ensure a total protein intake of 1.5–1.6 g/kg/day in the HIT-RT and 1.2–1.3 g/kg body mass/day (Bauer et al., 2013) in the CG. The chemical score of the protein product is 159. A hundred grams of powder contained 80 g of (whey) protein, with high l-leucine (9 g) and essential amino acid (57 g) components and 1,200 mg of calcium.

### Vitamin D and Calcium Supplementation

The participants were supplemented with cholecalciferol (MYVITAMINS, Manchester, UK) based on their serum 25OH vitamin D 3 (25OH D3) levels (ECLIA; Roche Diagnostics, Penzberg, Germany). The participants with serum concentrations below 75 nmol/L (*n* = 37) were provided with 10,000 IU/week, while the participants with 76 to ≤100 nmol/L were asked to take 5,000 IE/week.

We aimed to ensure a calcium intake of 1,000 mg/day in all the participants (DVO, 2017). Calcium intake was determined by dietary calcium questionnaires (Rheumaliga, Switzerland); deficiencies were compensated by calcium capsules (Sankt Bernhard, Bad Dietzenbach, Germany).

### Compliance With the Intervention

High emphasis was placed on monitoring participants’ compliance with the exercise protocol. Attendance rate and duration of the session were accurately determined by the gym’s chip card system. Based on validated predicting equations (Kemmler et al., 2008), we monitored the relationship of applied load and number of repetitions listed by the participants. A difference of ≥10% between the load selected by the participant and the load predicted by the equation was considered as inadequate effort.

Adherence to the prescribed supplementation of protein, cholecalciferol, and calcium was monitored by (a) checking our distribution logs, (b) biweekly phone calls, and (c) personal interviews conducted at FU assessments.

### Primary Study Outcomes

- Lean body mass changes (kg) as determined by dual-energy X-ray absorptiometry (DXA)
- Total body fat mass changes (kg) as determined by DXA
- Abdominal body fat percentage changes (%) as determined by DXA
- Maximum isokinetic hip‐ and leg-extensor strength (N) as determined by an isokinetic leg press

### Experimental Study Outcomes

- Differences between the 8-, 12-, and 18-month effects for:
  - Lean body mass
  - Total body fat mass
  - Abdominal body fat percentage
  - MILES

### Changes of Trial Outcomes After Trial Commencement

Due to a technical failure of the DXA scanner, body composition was determined after 8 months instead of 6 months.

### Assessments

The participants were required to maintain physical activities and diet and not exercise 48 h prior to the tests. All tests were consistently conducted and analyzed in our lab at the same time of the day (±2 h) in identical order and consistently with the same calibrated devices and by the same research assistant.

Body height was assessed by a Holtain stadiometer (Crymych Dyfed., Great Britain), and body mass was determined by the scale function of the Bio-Impedance-Analysis (DSM-BIA; InBody 770, Seoul, Korea) device. Body composition was evaluated by DXA (QDR 4500a, Discovery-upgrade, Hologic Inc., Bedford, MA, USA). Using the “compare mode,” the area and the placement of the baseline total body scans (excluding the skull) could be reproduced exactly during FU assessments. LBM was defined as soft fat-free mass (i.e., lean mass excluding the bone), and SMI was calculated as appendicular LBM mass divided by body height (kg/m^2^). Abdominal region of interest was specified as the area between the lower edge of the 12th rib and the upper edge of the crest iliac. Abdominal body fat percentage (i.e., abdominal fat rate) was defined as the rate of abdominal fat divided by total abdominal mass (i.e., sum of lean mass, including bone, and fat mass). The coefficient of variation (CV) and intra-class correlation coefficient (ICC) in our lab were ICC = 0.995 and CV = 0.5% for lean mass, ICC = 0.997 and CV = 1.2% for total body fat mass, and ICC = 0.994 and CV = 1.5% for abdominal fat percentage. The long-term CV of our DXA device averages 0.57% for the 18-month study period.

Maximum bilateral isokinetic hip/leg-extension strength was determined using an isokinetic leg press (CON-TREX LP, Physiomed, Laipersdorf, Germany). The tests were conducted in a sitting, slightly supine (15°) position, fixed by hip and chest straps. Using the standard velocity of 0.5 m/s, the range of motion within the knee angle was 30–90°. After briefing and a familiarization trial with low effort, the participants conducted five repetitions with maximum voluntary effort (“push as strongly as possible”). The higher value of two trials intermitted by 2 min of rest was included in the analysis. ICC and CV maximum bilateral isokinetic hip/leg-extension strength in this cohort were 0.990 (ICC) and 4.4% (CV).

In order to verify our predicting equation (Kemmler et al., 2008), we conducted 1RM-maximum tests (2s–1s–2s) for leg and bench press according to the approach of Kraemer et al. (1991).

All the participants were requested to complete a standardized questionnaire (Kemmler et al., 2004a) at baseline. We particularly asked for (a) demographic parameters, (b) diseases, pharmacologic therapy/dietary supplements, and hospitalization, (c) physical limitations, (d) falls and injurious falls, (e) injuries and low-trauma fractures within the last year, and (f) lifestyle, including physical activity and exercise (Kemmler et al., 2004b). During each FU, the participants answered questionnaires that focused on changes and events with a potential effect on our study endpoints. For that reason, the participants carefully listed their medications, supplements, and diseases at home before visiting our lab. In order to generate high consistency, completeness and accuracy, the primary investigator then checked the completed FU questionnaires in close interaction with the participants.

### Sample Size Analysis

The sample size calculation for the FrOST project was based on changes of BMD at the lumbar spine as the critical aspect of our research project on osteosarcopenia. However, applying reasonable assumptions transferred from a recent study on whole-body electromyostimulation in older men with sarcopenic obesity (Kemmler et al., 2017b, 2018) indicates that the present sample size generates sufficient statistical power (≥85%) to address the present research issue on LBM, body fat, and maximum hip/leg strength.

### Statistical Analysis

The primary study outcomes were calculated by intention-to-treat (ITT) analysis that included all participants randomly assigned to the study arms (HIT-RT vs. CG). R statistics software (R Development Core Team, Vienna, Austria), in combination with Amelia II, was used to conduct multiple imputation (ITT; Honaker et al., 2011). Using the full data set, imputation was repeated 100 times. Imputation diagnostic plots indicated that the imputation worked well. After checking for the normal distribution of data (Shapiro-Wilks, qq plots), all outcomes addressed here were analyzed by dependent *t*-tests. The *t*-test comparisons with pooled SD were applied to determine “effects,” defined as differences in changes of the given outcome between HIT-RT and CG. We consistently applied two-tailed tests; significance was accepted at *p* < 0.05. To adjust primary study outcomes for multiplicity, we used the Bonferroni-Holm method (Holm, 1979). Experimental study outcomes were analyzed by per-protocol analysis that included participants with all follow-up data (8, 12, and 18 months), independent of their compliance (HIT-RT: 18 vs. CG: *n* = 18). According to Li et al. (2017), the experimental endpoints were not adjusted for multiplicity. The standardized mean difference (SMD) according to Cohen (1988) was calculated to illustrate the effect sizes. Data analysis took place from July 2018 (baseline assessment) to January 2020 (18-month FU).

## Results

Table 1 lists the baseline characteristics of HIT-RT participants and CG. Some particularities should be noted. Firstly, total body fat data indicated a high prevalence of overweight and obesity in both groups. Baseline protein intake was high, particularly in the CG, and significantly differed from HIT-RT. Serum 25OH D3 was below the current recommendations (30 ng/ml; DVO, 2017) in all but four participants (CG: *n* = 3). Habitual gait velocity was above the sarcopenia cutoff criteria (0.8 m/s) in all but two subjects, while on average about half of the HIT-RT and CG fell within the sarcopenia criteria (<30 kg) for grip strength (Cruz-Jentoft et al., 2010).

**Table 1:** Baseline characteristics of the participants of the high velocity/intensity/effort progressive resistance training (HIT-RT) and the control group (CG).

Variable	HIT-RT (*n* = 21) MV ± SD	CG (*n* = 22) MV ± SD
Age (years)	77.8 ± 3.6	79.2 ± 4.7
Body mass index (kg/m^2^)	25.0 ± 3.0	24.5 ± 1.9
Total body fat (DXA; %)	34.5 ± 6.1	33.6 ± 4.0
Waist circumference (cm)	92.4 ± 10.3	89.2 ± 8.9
Skeletal muscle mass index (kg/m^2^)^a^	6.89 ± 0.31	7.01 ± 0.27
Habitual gait velocity (m/s)	1.25 ± 0.17	1.26 ± 0.15
Hand grip strength (kg)	30.7 ± 5.1	30.0 ± 4.3
Multimorbidity (*n*)^b^	10	12
Hand or lower limb arthritis (*n*)	3	3
Physical activity (index)^c^	4.45 ± 1.32	4.15 ± 1.53
Training volume (min/week)	46 ± 52	59 ± 56
25OH D3 (ng/ml)^d^	21.6 ± 8.4	17.5 ± 7.0
Calcium intake (mg/d)^e^	802 ± 226	833 ± 282
Energy intake (kcal/d)^f^	2,155 ± 416	2,291 ± 590
Protein intake (g/kg/d)^f^	1.10 ± 0.25	1.29 ± 0.34

MV, mean values; SD, standard deviation.

a Appendicular skeletal muscle mass/body mass^2^.

b Two or more diseases based on the disease cluster of Schäfer et al. (2010).

c Scale from “very low” (1) to “very high” (7; Kemmler et al., 2004b).

d Roche Diagnostics, Mannheim, Germany.

e Calcium questionnaire (Rheumaliga, Switzerland).

f As determined by dietary records.

Three participants of the CG and two participants of the HIT-RT group were lost to 18-month follow-up (Figure 1). In summary, the participants of HIT attended 95 ± 5% of the exercise sessions. The duration of an exercise session varied between 35 and 50 min. Comparing individually selected loads and loads calculated by the predicting equation, we estimate that up to one-third of the RM sets were conducted with too low effort, particularly during phases 3 and 4. We did not observe any unintended side effects or injuries during the regular exercise sessions. However, one participant reported temporary worsening of an existing knee and shoulder pain during and after the exercise sessions. Compliance with protein, calcium, and cholecalciferol supplementation according to biweekly phone calls, supply logs, and personal interviews at follow-up assessments was considered as high. This estimation was particularly confirmed by our supply logs that determined an overlap of 82–94% for calcium, vitamin D, and protein between the prescribed doses and the amount of supplements requested by the participants during the 18-month study period.

### Study Endpoints

Table 2 displays the results of the primary endpoints. LBM increased significantly (*p* < 0.001) in the HIT-RT group and decreased non-significantly (*p* = 0.11) in the CG. Differences between the groups were significant (*p* < 0.001); the corresponding effect size was high (SMD: 1.26).

**Table 2:** Baseline data and changes of study outcomes in the HIT-RT and CG.

	EG MV (95% CI)	CG MV (95% CI)	Difference MV (95% CI)	*p*
Lean body mass (kg)				
Baseline	44.93 (42.81 to 47.06)	43.19 (40.99 to 45.39)	1.75 (−1.21 to 4.71)	0.239
Changes	1.47 (1.14 to 1.80)	−0.26 (0.06 to −0.57)	1.73 (1.13 to 2.32)	<0.001
Total body fat mass (kg)				
Baseline	24.16 (20.92 to 27.40)	21.95 (21.24 to 24.86)	2.22 (𥈒1.38 to 5.82)	0.221
Changes	−1.80 (−0.98 to −2.63)	0.64 (−0.17 to 1.46)	2.44 (1.28 to 3.60)	<0.001
Abdominal body fat percentage (%)				
Baseline	37.67 (34.86 to 40.48)	37.64 (34.60 to 40.68)	0.03 (−3.99 to 4.03)	0.990
Changes	−1.84 (−1.15 to −2.54)	0.84 (0.16 to 1.50)	2.68 (1.70 to 3.66)	<0.001
Maximum isokinetic hip leg-extensor strength (MILES; *N*)				
Baseline	1,620 (1,399 to 1,841)	1,746 (1,569 to 1,924)	126 (−138 to 402)	0.368
Changes	545 (459 to 641)	12 (−79 to 102)	533 (397 to 670)	<0.001

MV, mean value; CI, confidence interval.

Total body fat mass decreased significantly in the HIT-RT group (*p* < 0.001) and increased non-significantly (*p* = 0.12) in the CG. Group differences were significant (*p* < 0.001); effect size was high (SMD: 1.33). In parallel, abdominal body fat percentage decreased significantly in the HIT-RT group (*p* < 0.001) and increased significantly in the CG (*p* = 0.017). The differences between HIT-RT and CG were significant (*p* < 0.001; SMD: 1.71; Table 2).

Lastly, maximum strength increased significantly in the HIT-RT group (*p* < 0.001) and was maintained in the CG (*p* = 0.80). Group differences were significant (*p* < 0.001; SMD: 2.45; Table 2).

After adjusting for multiple testing with the Bonferroni-Holm method (Holm, 1979), all group differences remained highly significant (*p* < 0.001). Thus, all the primary hypotheses could be accepted.

**Figure 2:**
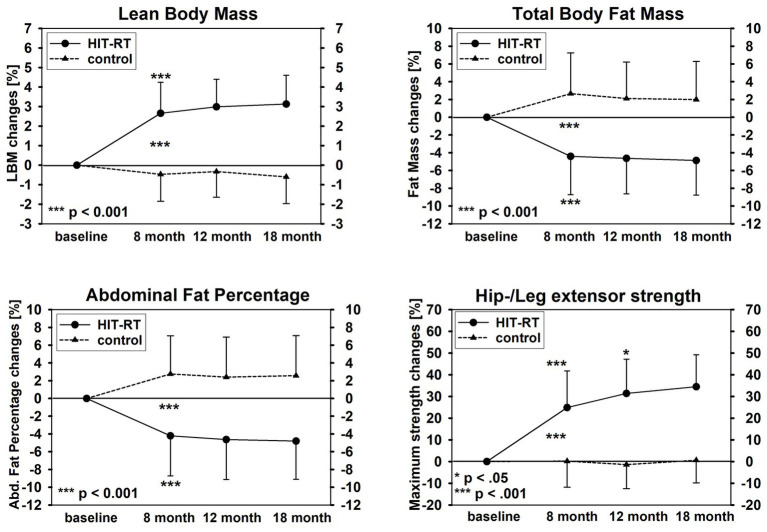
Changes of lean body mass (upper left graph), total body fat mass (upper right graph), abdominal fat percentage **(lower left graph)**, and maximum isokinetic hip/leg-extensor strength (lower right graph).

Table 3 and Figure 2 display the results on our experimental hypothesis (per-protocol analysis). After a significant initial effect from baseline to 8-month FU (Table 3), all in favor of the HIT-RT group, apart from a significant effect for MILES (8–18 months: *p* = 0.044; Figure 2, lower right graph), no further significant effects were determined for LBM (Figure 2, upper left graph), total body fat (Figure 2, upper right graph) or abdominal body fat (lower left graph) after month 8 (Table 3).

**Table 3:** Mean values and 95% CI of differences (absolute changes) between HIT-RT and control group for 8-, 12-, and 18-month assessments.

Variable	8-month MV (95% CI)	12-month MV (95% CI)	18-month MV (95% CI)
Lean body mass (kg)	1.47 (0.96–1.99)^a^	1.45 (1.00–1.90)	1.64 (1.17–2.09)
Total body fat (kg)	2.02 (1.21–2.82)^a^	2.09 (1.34–2.95)	2.27 (1.52–3.03)
Abdominal body fat (%)	2.33 (1.85–2.82)^a^	2.38 (1.63–3.13)	2.66 (1.67–3.66)
Maximum isokinetic hip/leg-extensor strength (*N*)	407 (248–565)^a^	512 (365–659)	534 (412–657)^b^

a Significant difference to previous assessment.

b Significant difference to 8-month assessment. Data were analyzed by per-protocol analysis.

When concentrating on the HIT-RT group, all parameters improved after month 8 (LBM/fat mass/−rate: ≈1–2% to MILES: 7%); however, significant changes were recorded only for MILES at 12/18 months (*p* = 0.037 and 0.012; Figure 2).

### Confounding Parameters

25OH D3 increased in both groups (HIT-RT: 28.1 ± 6.1 vs. CG: 29.6 ± 5.8 ng/ml); however, 11 participants were each below current recommendations (DVO, 2017). Dietary intake parameters (e.g., energy, protein, and calcium) did not differ significantly (*p* > 0.430) between the FU assessments. In parallel, significant changes of habitual physical activity (*p* > 0.687) and exercise outside FrOST were not reported. Two men of the HIT-RT group and one man of the CG listed longer periods (3–5 weeks) of inactivity due to diseases or hospitalization.

## Discussion

The primary aim of FrOST was to address recognized parameters of osteoporosis and sarcopenia by HIT-RT (and dietary supplements) in older men with osteosarcopenia. In order to determine the full amount of exercise-induced changes of bone mineral density (Eriksen, 2010), we initiated a long intervention/study period that now enables us to determine the long-term effect of a periodized HIT-RT on body composition and strength in this cohort of older men. Applying exercise intensity peaks in the range corresponding to 60–85% 1RM, high velocity, and RM with intensifying strategies (Gießing, 2008), most determinants responsible for initiating a hypertrophic response (Schoenfeld, 2010) were considered by our protocol. In summary, we observed significant favorable effects on LBM, total and abdominal fat, and lower extremity strength (MILES) that were superior to current studies on sarcopenic/SO cohorts, be it with or without protein supplementation (review in Martinez-Amat et al., 2018; Vlietstra et al., 2018; Hsu et al., 2019). Closest to our study, after 8 months of multiple-set DRT on machines (+whey protein supplementation) applied either two or three times/week, Stec et al. (2017) reported LBM changes of 0.4–1.89 kg in older people (60–75 years) with age-related muscle atrophy. While all four subgroup protocols focus on hypertrophy (three sets of eight to 12 reps to “failure”), the most successful protocol (*p* < 0.05 to other groups) on LBM scheduled two sessions with work to “failure” and one session/week with nRM. In parallel, 1RM leg-press (≈MILES) increased similarly in all subgroups by 40–55% (FrOST: 34%). Total body fat mass decreased by about 3–5% (FrOST: 7.5%). However, apart from the longer duration, the main differences to the present studies was our time-effective (<50 min/session), single-set approach applied only two times/week. We observed a very high attendance rate (95%), i.e., our exercise protocol was not only effective but obviously feasible and attractive even in this cohort with a low affinity to DRT (Carlson et al., 2010). Apart from its time effectiveness, another reason for the attractiveness of our approach might be the close involvement and interaction with the participants. Nevertheless, the participants did not always respect the “effort” specification prescribed by the training logs. Although the number of RM set conducted with obviously too low effort tended to decrease during the intervention, this “limitation” remained up to the study end. On the other hand, the aspect that about 60–70% of the RM sets were conducted at least with adequate effort can be considered as a satisfying result for a non-athletic cohort. Of note is that noncompliance with exercise intensity is rarely reported by DRT trials, although inadequate repetition to load rate might be the main reason for a lack of hypertrophic effects (Schoenfeld, 2012, 2013).

In FrOST, we clearly determined the significant positive effect of HIT-RT on total and abdominal body fat. Although there is evidence for the body-fat-reducing effect of RT (Strasser and Schobersberger, 2011), the finding that reductions of body fat exceed increases of LBM could not have been necessarily expected. One may speculate that, apart from the acute energy expenditure and a prolonged period of energy demands due to muscular synthesis, repair, and adaptation after intense muscular loading (Teschler et al., 2018), increased resting metabolic rate that largely depends on muscle mass might be the dominant trigger for fat reduction (Strasser and Schobersberger, 2011; Strasser et al., 2012). However, considering the development of LBM (Figure 2), the non-time-delayed decline and “stagnation” of total and abdominal fat did not support this estimation. Apart from HIT-RT, there is significant evidence that high-protein diets generate favorable effects on parameters closely related to obesity parameters. This includes specific effects on appetite, hunger, and satiety hormones (Bendtsen et al., 2013; Chungchunlam et al., 2015), significant enhancement of fat oxidation, and increased thermogenesis (Acheson et al., 2011; Gentile et al., 2015). Although studies that aimed to determine the effects of whey protein on fat reduction are rare and inconsistent, some of them revealed positive results. As an example, after 23 weeks of whey protein supplementation (56 g/day) in overweight–obese adults, Baer et al. (2011) reported a significant reduction of 2.3 kg in fat mass and 2.4 cm in waist circumference compared with an isoenergetic carbohydrate supplementation.

Although our DRT is not a purebred HIT-RT since we did not include the momentary failure approach characteristic for HIT-RT (Gießing, 2008), high-velocity/intensity/effort protocols might be considered as inadequate or even dangerous for older cohorts. We do not agree with this undifferentiated view. Firstly, high velocity during resistance exercises is reported to be well applicable and tolerable for the older subject (Liu and Latham, 2009; Mangione et al., 2010). Furthermore, considering (1) our careful conditioning period, (2) continuous supervision by certified trainers, (3) the periodized protocol with “active regeneration” weeks, (4) sets with explosive movements not conducted to RM, (5) detailed briefing in joint meetings, and (6) a reasonable mix of nRM and RM sets, we regard FrOST as a blueprint for an effective, attractive, and safe DRT program for older people.

Unfortunately, our study design, limited statistical power, and the delay of the intended 6-month FU prevent a decisive conclusion about the ongoing effects of a periodized state-of-the-art HIT-RT on body composition and strength parameters. Nevertheless, whether sophisticated exercise protocols are worth the effort, at least in non-athletic cohorts, is an important, currently unaddressed issue. Our results indicated continuing, albeit only small to moderate, further effects on body composition and strength (Table 3). We are unable to validly attribute those changes to our dedicated exercise program. However, we speculate that less-progressive exercise programs, e.g., protocols that simply adapt load to the increased performance of their participants might fail to demonstrate ongoing significant effects even on maximum strength after 8 or 12 months of exercise. Another less physiological aspect might be of prominent importance in this context. Considering that it is not always easy for our older, less sportive seniors to reliably join exercise programs, we should be aware of our responsibility to create the best possible program for these participants, although trainers and staff may sometimes argue that less effort might generate similar effects.

Some study features and limitations should be addressed to allow the reader to properly interpret our results: (1) we applied adequate protein, cholecalciferol, and calcium supplementation for both groups (Bauer et al., 2013; DVO, 2017). In accordance with recent recommendations (Bauer et al., 2013), higher doses were scheduled for the exercisers. Thus, although our cohort with adequate dietary intakes (Table 1) might benefit less from additional protein supplementation (Dalle et al., 2017), the higher total protein intake of the HIT-RT group might have contributed to our results. Nevertheless, evidence for an additional effect of protein to DRT on muscle mass or strength in older people with sarcopenia or SO is limited (Liao et al., 2017a; Hita-Contreras et al., 2018; Martinez-Amat et al., 2018; Vlietstra et al., 2018; Beckwee et al., 2019; Hsu et al., 2019); (2) our eligibility criteria for sarcopenia focus on skeletal muscle mass index; functional aspects were not considered. We determined “skeletal muscle mass” (or more precisely “soft lean body mass”) by DXA technique. Although a large variety of techniques are available to assess body composition (e.g., MRI, BIA, CT, and creatinine dilution), a recent expert panel recommended DXA as the standard reference for measuring muscle mass (Buckinx et al., 2018). However, some limitations of this technique should be considered. Besides the possibility that hydration status in particular can affect the results on LBM, the main limitation of DXA might be its inability to separate different soft tissue components (e.g., muscle vs. organs), which prevents a proper assessment of trunk muscle mass. Further skeletal muscle fat infiltration cannot be determined by DXA; (3) a technical failure of the DXA scanner prevented body composition assessment after 6 months as intended; (4) accepting that BMI is an inadequate parameter to classify overweight and obesity in sarcopenic people and applying an obesity cutoff of 27–30% total body fat, as suggested by the majority of studies (Donini et al., 2019), the vast majority (75–94%) of our cohort was “osteosarcopenic obese” (Bauer et al., 2019). This high potential might contribute to explaining the pronounced decreases in total and abdominal body fat; and (5) due to the mandatory demands of RT devices and careful supervision, the present HIT-RT approach cannot be transferred into a home-based training protocol. However, the large number of commercial and non-commercial providers in the area of health-orientated DRT might easily allow the broad implementation of this highly efficient DRT protocol. Nevertheless, one limitation, though restricted to Germany, has to be considered in this context. “Rehabilitation sport” according to German law (SGB_IX, 2019), the primary vehicle of secondary and tertiary prevention of chronic diseases by means of exercise, explicitly prohibits the application of resistance exercise devices. Considering the safety and the effectiveness of this type of DRT, we think that this specification ought to be revised in the nearest future.

In conclusion, the present HIT-RT/dietary supplement approach was an effective, attractive, feasible, and safe vehicle to improve body composition and muscle strength in older community-dwelling men with (osteo-)sarcopenia. Considering that sarcopenia and sarcopenic obesity demonstrate aspects (Calvani et al., 2013; Sullivan-Gunn and Lewandowski, 2013; Kob et al., 2015) negatively related to muscle protein synthesis (Dalle et al., 2017), we speculate that HIT-RT-induced changes in muscle mass and strength might be even more prominent in healthy older people.

## Data Availability Statement

The raw data supporting the conclusions of this article will be made available by the authors, without undue reservation.

## Ethics Statement

The studies involving human participants were reviewed and approved by Friedrich Alexander University Erlangen-Nürnberg Ethics Committee (number 67_15b and 4464b). The patients/participants provided their written informed consent to participate in this study.

## Author Contributions

WK, MK, SS, and DS designed the study, completed the data analysis and/or interpretation, and drafted the manuscript. MF and KE contributed to the study conception and design and revised the manuscript. WK accepts responsibility for the integrity of the data sampling, analysis, and interpretation. All authors contributed to the article and approved the submitted version.

### Conflict of Interest

The authors declare that the research was conducted in the absence of any commercial or financial relationships that could be construed as a potential conflict of interest.
